# Pathophysiological Mechanisms of Peritoneal Fibrosis and Peritoneal Membrane Dysfunction in Peritoneal Dialysis

**DOI:** 10.3390/ijms25168607

**Published:** 2024-08-07

**Authors:** Yasuhiko Ito, Ting Sun, Mitsuhiro Tawada, Hiroshi Kinashi, Makoto Yamaguchi, Takayuki Katsuno, Hangsoo Kim, Masashi Mizuno, Takuji Ishimoto

**Affiliations:** 1 Department of Nephrology and Rheumatology, Aichi Medical University, Nagakute 480-1195, Japankinashi.hiroshi.909@mail.aichi-med-u.ac.jp (H.K.); yamaguchi.makoto.231@mail.aichi-med-u.ac.jp (M.Y.); takuji.ishimoto@aichi-med-u.ac.jp (T.I.); 2 Department of Nephrology, Imaike Jin Clinic, Nagoya 464-0850, Japan; 3 Department of Nephrology and Rheumatology, Aichi Medical University Medical Center, Okazaki 444-2148, Japan; t-katsuno@aichi-med-u.ac.jp; 4 Department of Nephrology, Nagoya University Graduate School of Medicine, Nagoya 466-8550, Japan; kimu13@med.nagoya-u.ac.jp (H.K.); mizuno.masashi.z6@f.mail.nagoya-u.ac.jp (M.M.)

**Keywords:** peritoneal fibrosis, inflammation, peritoneal membrane dysfunction, D/P Cr, PD dialysate, EPS

## Abstract

The characteristic feature of chronic peritoneal damage in peritoneal dialysis (PD) is a decline in ultrafiltration capacity associated with pathological fibrosis and angiogenesis. The pathogenesis of peritoneal fibrosis is attributed to bioincompatible factors of PD fluid and peritonitis. Uremia is associated with peritoneal membrane inflammation that affects fibrosis, neoangiogenesis, and baseline peritoneal membrane function. Net ultrafiltration volume is affected by capillary surface area, vasculopathy, peritoneal fibrosis, and lymphangiogenesis. Many inflammatory cytokines induce fibrogenic growth factors, with crosstalk between macrophages and fibroblasts. Transforming growth factor (TGF)-β and vascular endothelial growth factor (VEGF)-A are the key mediators of fibrosis and angiogenesis, respectively. Bioincompatible factors of PD fluid upregulate TGF-β expression by mesothelial cells that contributes to the development of fibrosis. Angiogenesis and lymphangiogenesis can progress during fibrosis via TGF-β–VEGF-A/C pathways. Complement activation occurs in fungal peritonitis and progresses insidiously during PD. Analyses of the human peritoneal membrane have clarified the mechanisms by which encapsulating peritoneal sclerosis develops. Different effects of dialysates on the peritoneal membrane were also recognized, particularly in terms of vascular damage. Understanding the pathophysiologies of the peritoneal membrane will lead to preservation of peritoneal membrane function and improvements in technical survival, mortality, and quality of life for PD patients.

## 1. Introduction

Peritoneal dialysis (PD) is a renal replacement therapy that is widely used worldwide due to the good outcomes. In Hong Kong and Thailand, penetration of this therapy is high due to national policies of PD-first [1]. The advantages of PD over hemodialysis include the following: (1) hemodynamic stability during treatment, prolonging the preservation of residual renal function; (2) ease of lifestyle adjustment; and (3) the ability to perform treatment at home, which is particularly beneficial for elderly patients. However, PD has the disadvantages of complications including peritonitis and limited treatment duration due to peritoneal membrane dysfunction. Treatment duration for PD is longer in Japan due to the low prevalence of kidney transplantation [2,3]. Long-term PD is associated with peritoneal dysfunction, which may be one cause of discontinuation from PD [4,5]. In addition, patients with ultrafiltration (UF) failure (UFF) and fluid overload display poor survival rates [6,7,8].

Many pathological factors in the peritoneal membrane affect peritoneal membrane transport rates and net UF, including blood vessels, lymphatic vessels, and mesothelial cells, fibrosis with extracellular matrix (ECM) and fibroblasts, monocytes/macrophages, neutrophils, mast cells, T lymphocytes, and dendritic cells, which can produce chemokines and cytokines and are involved in immune responses [9,10,11]. As a result, peritoneal pathological fibrosis proceeds and leads to UFF.

In the microvasculature of the peritoneal membrane, blood is generally supplied from a small artery to the capillaries. Capillaries represent the main site of solute and fluid exchange, and dialysate containing glucose is absorbed through capillaries. Post-capillary venules are primary sites for exposure of glucose-containing dialysate and for leukocyte adhesion, and they show the most significant changes in pathology and permeability [10]. These structures are damaged during treatment with PD.

This review focuses on the pathogenesis of peritoneal membrane injury, fibrosis and related pathologies, and peritoneal membrane dysfunction in PD including lymphangiogenesis and complement activation, which have not been discussed in detail before. We also summarize and discuss the time course of events and factors responsible for alterations to the structure and function of the human peritoneal membrane.

## 2. History and Characteristics of Human Peritoneal Membrane Alterations in PD

Peritoneal pathology differs markedly between humans and rodents. In addition, establishment of an accurate model of the chronic peritoneal injury seen in human PD treatment has been difficult. The study of biopsied peritoneal membrane from humans is therefore important.

Findings of human healthy peritoneal membrane were reported in 2016 [12]. A total of 106 normal peritoneal tissues from patients 0.1–60 years old were examined. The results showed that peritoneal thickness was greatest between 7 and 12 years old, and blood capillary density was greatest at less than 1 year old, lowest between 7 and 12 years old, and increased with age. Lymphatic vessel density is also highest at less than 1 year old but decreases with age.

Changes in peritoneal tissue due to PD were first reported in 1981 [13]. Peritoneal tissues from 16 patients treated with PD were examined, revealing the presence of more fibrosis compared to patients with uremia or HD [14]. Paolo et al. examined peritoneal capillaries and mesothelium and found basement membrane thickening and marked replication of mesothelial cells after several months of PD in both diabetes mellitus and non- diabetes mellitus patients [15]. Vascular injury in patients receiving continuous ambulatory peritoneal dialysis was evaluated using immunofluorescence microscopy and electron microscopy, revealing collagen fiber deposition and smooth muscle cell degeneration [16]. An evaluation of peritoneal tissues from 25 German PD patients found that peritoneal thickening was related to vessel number and vascular damage, and identified a correlation between the dialysate-to-plasma ratio of creatinine (D/P Cr) and cumulative glucose exposure [17].

Williams in 2002 [18] and Honda in 2008 [10] summarized methods for assessing peritoneal membrane tissues, leading to much more accurate assessments and comparison of peritoneal tissue studies in large cohorts of patients with long-term PD. Williams et al. examined the peritoneum of 130 PD patients and found that peritoneal thickening and vasculopathy increased with PD duration, and the number of vessels increased in patients with membrane failure [18]. They also compared the peritoneal tissues of 157 PD patients with findings at baseline and reported that peritoneal thickening and vascular damage progressed with PD duration [19].

Around 2008, many researchers reported that peritoneal membrane biopsy showed a thickened peritoneal membrane after treatment with PD [20,21,22]. In this period, most patients with PD were treated using conventional PD fluid (PDF). Vessel injury with vasculopathy was observed in all studies [20,21,22]. Vessel numbers and vascular surface area tended to be increased, but no significant differences in duration of PD or vessel number were observed [19,21,23]. Numata et al. indicated that increased vascularization and/or dilatation of vessels correlated with a high peritoneal solute transport rate [23]. A variant in aquaporin-1 (AQP1) was recently reported to be associated with peritoneal UF volume and related to mortality [24]. This important discovery reveals a mechanism that is related to the variability in the UF volume at the initiation of PD. Interestingly, expression of AQP1 was significantly higher in vascular endothelial cells from patients with peritoneal membranes > 400 μm thick, and a correlation was found between free water transport and AQP1 expression [25]. In contrast, net UF and sodium sieving were closely and inversely correlated with submesothelial thickness and collagen volume fraction in the submesothelial area, although AQP1 expression was unchanged in another study [26]. Unfortunately, genetic variation and collagen volume fraction and density were not evaluated in all cases.

High levels of glucose in peritoneal dialysate were reported to cause progressive deposition of advanced glycation end products (AGEs) in the peritoneum. Strong staining for AGEs was evident in mesothelial cells, vessel walls, and connective tissues, and correlated with increased permeability of the peritoneal membrane [27]. In addition, a comparison of low- and high-UF groups of 14 PD patients each showed inverse correlations between UF volume and interstitial fibrosis, microvascular sclerosis, and microvascular AGE deposition [28].

In a recent report, AGE accumulation in the interstitium and vessel walls correlated positively with the use of conventional PDF. D/P Cr and AGE accumulation in the vessel wall correlated with immature capillary density [29]. The AGE-expressing area is now considered a marker of peritoneal membrane injury [30].

In 2020, the Japanese Society for Peritoneal Dialysis (JSPD) published a position paper on peritoneal tissue sampling methods, as accurate morphological assessment of peritoneal tissues is often difficult due to scarring around the PD catheter insertion site, differences in sampling sites, and variability in supporting connective tissue including fascia. The JSPD thus recommends methods of harvesting parietal peritoneum (1.5 × 1.5 cm in size) and the posterior sheath of the rectus abdominis muscle at a point 3 cm below the site of PD catheter insertion during catheter removal [31]. Standardization of collection methods and sites will enable more accurate assessment and better interpretation of peritoneal pathologies.

## 3. Peritoneal Membrane Dysfunction and Pathological Changes in PD: Roles of Fibrosis, Angiogenesis, and Lymphangiogenesis

The characteristic feature of chronic peritoneal damage in PD treatment is a loss of UF capacity associated with pathological fibrosis and neoangiogenesis based on histopathological findings from human peritoneal membrane and animal experiments [9,10,18].

**Figure 1:**
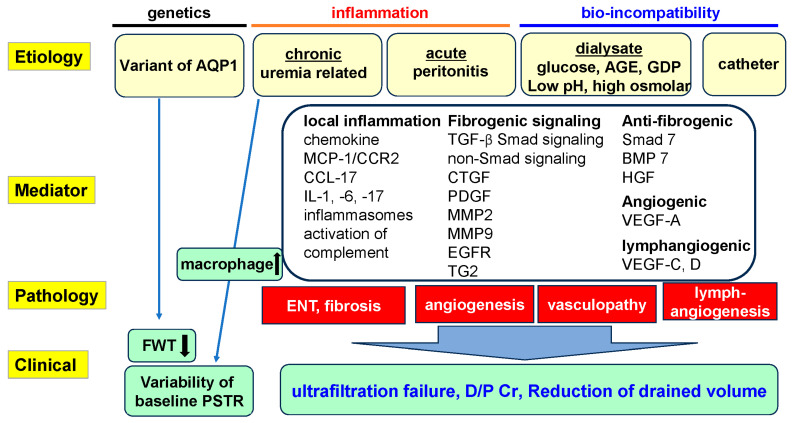
Summary of the etiology, mediators, and pathologies responsible for peritoneal membrane changes and dysfunction. The pathogenesis of peritoneal fibrosis is attributed to a combination of bioincompatible factors including AGEs, GDPs, pH, and high glucose concentration in PDF and catheter, along with peritonitis (particularly recurrent episodes of peritonitis). Uremia is associated with inflammation of the peritoneal membrane before PD initiation. AQP1 variants and peritoneal inflammation are related to variability in the UF volume at initiation of PD. Several kinds of mediators are involved in inducing phenotypic changes in mesothelial cells as epithelial–mesenchymal transition, leading to fibrosis associated with angiogenesis and lymphangiogenesis. Vasculopathy develops, particularly with the use of acidic conventional solution. UFF and peritoneal membrane dysfunction progress over time, particularly in patients (except children) treated with conventional PDF. AGEs: advanced glycation end products; AQP1: the gene that encodes aquaporin-1, GDPs: glucose degradation products; BMP7: bone morphogenetic protein 7; CCL: C-C motif chemokine ligand; CCR2: C-C chemokine receptor 2; CTGF: connective tissue growth factor; D/P Cr: dialysate-to-plasma ratio of creatinine; EGFR: epidermal growth factor receptor; FWT: free water transport; HGF: hepatocyte growth factor; IL: interleukin; MCP-1: monocyte chemoattractant protein-1; MMPs: matrix metalloproteinases; PDGF: platelet-derived growth factor; PSTR: peritoneal solute transfer rate; Smad: Suppressor of mothers against decapentaplegic; TGF-β: transforming growth factor-β; TG2: transglutaminase 2; VEGF: vascular endothelial growth factor. ↑: increase, ↓: decrease.

The pathological condition of the peritoneal membrane is usually assessed by the presence or absence of mesothelial cells, peritoneal thickening, the submesothelial compact zone including fibroblasts and collagen fibers, number of vessels, and the extent of vascular damage (vasculopathy) [10,32]. The pathogenesis of peritoneal fibrosis is attributed to a combination of bioincompatible factors, including AGEs, glucose degradation products (GDPs), low pH and high glucose in PDF, and peritonitis, particularly with recurrent episodes of peritonitis. Uremia is associated with inflammation of the peritoneal membrane even before PD initiation [33] (Figure 1). These factors induce phenotypic changes in mesothelial cells as epithelial–mesenchymal transition (EMT), leading to fibrosis associated with angiogenesis. In addition, lymphangiogenesis is thought to be involved in net UF [34,35,36,37].

### 3.1. Angiogenesis, Peritoneal Fibrosis, and Vasculopathy

#### 3.1.1. Peritoneal Fibrosis and Angiogenesis

In the analysis of peritoneal thickening, the submesothelial compact zone is identified and its thickness is measured at five points, then the average value is calculated. With the use of PD solutions, parietal peritoneal thickness and the number of vessels progressively increase with the duration of PD treatment [10,32,38]. In rat models, an inverse correlation was seen between the number of peritoneal blood vessels and net UF after 4 weeks of daily infusion with conventional PDF [37], indicating that PDF represents a factor in the induction of angiogenesis and peritoneal fibrosis (Figure 1). Neoangiogenesis increases the vascular surface area, which is related to solute transport. On the other hand, fibrosis plays a role in the resistance to water flow [39]. Inflammation induces neoangiogenesis and fibrosis and increases permeability. Increased peritoneal permeability is largely induced by neoangiogenesis and fibrosis. Changes in the human peritoneal membrane are primarily described in Section 5.

#### 3.1.2. Relationship between Peritoneal Fibrosis and Angiogenesis

The reasons neoangiogenesis develops in association with fibrosis have remained unclear. Exploration of these mechanisms is important to identify whether fibrosis or neoangiogenesis should be the main target of treatments for UFF. In addition, interactions between transforming growth factor (TGF)-β, as the main mediator of fibrosis, and vascular endothelial growth factor (VEGF)-A, as the main mediator for neoangiogenesis in the peritoneum, were unclear. We therefore investigated these mechanisms.

In both 4 h and overnight-dwelling human PDF, VEGF-A and TGF-β1 levels in PD effluent correlated with the D/P Cr [40]. In addition, a correlation was identified between TGF-β and VEGF-A. Analyses of human peritoneal membrane tissues showed that VEGF-A mRNA levels in the peritoneal membrane were significantly higher in patients with UFF. VEGF-A expression correlated with the number of CD-31-positive vessels and the thickness of the submesothelial compact zone, suggesting that neoangiogenesis is linked to peritoneal fibrosis [40].

In an animal model of chlorhexidine gluconate-induced peritoneal fibrosis, inhibition of TGF-β receptor inhibitor reduced fibrosis, VEGF-A expression, and CD-31-positive vessels, indicating that inhibition of fibrosis reduces neoangiogenesis. In an in vitro study, TGF-β1 induced VEGF-A production in both cultured human peritoneal mesothelial cells (HPMCs) and cultured fibroblasts [40]. These data indicate that TGF-β1 directly induces VEGF-A production in both mesothelial cells and fibroblasts. TGF-β also augments inflammation- or hypoxia-induced VEGF-A production in macrophages [41,42]. Such findings indicate a direct link between TGF-β and VEGF-A (the TGF-β–VEGF-A pathway) resulting in fibrosis being associated with neoangiogenesis. In addition, high glucose-induced pseudohypoxia is a characteristic feature of treatment with PD. This condition stimulates production of hypoxia-inducible factor (HIF)-1, leading to upregulation of TGF-β, VEGF, plasminogen activator inhibitor 1, and connective tissue growth factor/cellular communication network factor 2 (CTGF/CCN2) [43,44]. Fibrosis (TGF-β) could thus represent an optimal target for blocking not only fibrosis, but also neoangiogenesis.

#### 3.1.3. Vasculopathy

Vasculopathy pathologically indicates damage to the vascular endothelial cells and is an important factor in peritoneal membrane solute transport (Figure 1). Vasculopathy is assessed at the level of the post-capillary venule (PCV) as the ratio of luminal diameter (L) to vessel diameter (V), termed the L/V ratio, because the PCV is the site most exposed to dialysate and tends to show significant damage [10,32].

As PD treatment is prolonged, vascular narrowing as assessed by the L/V ratio becomes worse in patients treated with conventional PDF [10,32,45] (Figure 1). Differences in peritoneal membrane pathology between patients using conventional PDF and those using low-GDP pH-neutral solution (pH-neutral PDF) are described in Section 5.

### 3.2. Major Factors Inducing Peritoneal Fibrosis and Injury

#### 3.2.1. Peritonitis

Peritonitis, particularly if prolonged, severe, or recurrent, often causes inflammation, fibrosis, and neoangiogenesis [46] (Figure 1). Early diagnosis and appropriate therapy for peritonitis are thus important to maintain peritoneal membrane function. The main mediators of fibrosis and neoangiogenesis are TGF-β and VEGF-A, respectively. Details are described in Section 3 below. Infectious and non-infectious models of peritonitis-induced peritoneal injury for research are described elsewhere [46].

#### 3.2.2. AGEs as Bioincompatible Factors in PDF

Accumulation of uremic toxins, sodium, AGEs and asymmetric dimethylarginine, and the bioincompatible characteristics of dialysate can trigger the development of inflammation [47,48] (Figure 1). Heat with autoclave sterilization of conventional glucose-based PDF causes elevated levels of dialysate GDPs. Under conditions of elevated glucose, AGEs form from the non-enzymatic glycosylation of proteins. These factors contribute to the bioincompatibility of dialysate (Figure 1).

AGEs are reported to accumulate in the peritoneal membrane, including in the interstitium and vessel walls. AGE accumulation increases with prolonged PD treatment and the extent of vascular sclerosis correlates with the degree of vascular AGE accumulation [27,28,45]. Exposure to GDPs has been shown to induce mesothelial cell and vascular endothelial cell injuries [49,50,51,52]. AGE deposition can be a factor inducing vasculopathy.

### 3.3. Relationship between Peritoneal Fibrosis and Lymphangiogenesis

**Figure 2:**
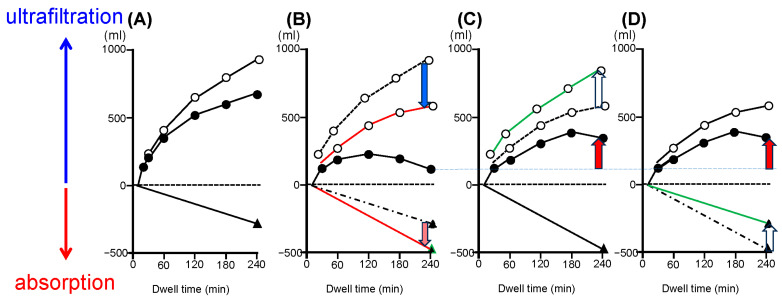
Determinants of ultrafiltration volume in PD. (**A**) Normal: Net ultrafiltration volume (black circle) is defined as transcapillary water transport (white circle) minus lymphatic absorption (black triangle). (**B**) UFF: After severe peritonitis, net ultrafiltration (black circle) decreases with a decline in transcapillary water transport (red line with blue arrow) and an increase in lymphatic absorption (red line with red arrow). (**C**) Suppression of angiogenesis against ultrafiltration failure: Suppression of angiogenesis (green line with white arrow) increases the drained volume (red arrow). (**D**) Suppression of lymphangiogenesis against ultrafiltration failure: Suppression of lymphangiogenesis (green line with white arrow) increases the drained volume (red arrow). Black dashed line indicates the baseline (ultrafiltration volume = 0 mL). Blue dashed line indicates the ultrafiltration volume at 240 min of dwelling time of (**B**).

The structure of lymphatic vessels is quite different from that of blood capillaries. Blood capillaries have a continuous basal lamina with tight interendothelial junctions and are surrounded by pericytes and smooth muscle cells. In contrast, lymphatic capillaries are characterized by a thin wall with wide lumen and a lack of pericytes or basement membrane. This structure allows easy absorption of fluid, cells, and macromolecules from the interstitium. Lymph is then further drained into collecting lymphatic vessels and returned to the circulation through the thoracic duct [37,53,54,55]. Absorption of lymph is expected to be increased in UFF (Figure 2), but results from human studies using radioactive tracers have been controversial [56,57] and the pathophysiology was unclear. We have conducted human, animal, and in vitro studies [35,37].

VEGF-C concentrations in human PD effluent dialysate correlate with the D/P Cr and the concentration of TGF-β1 in dialysate [53,58]. VEGF-C, -D, and markers for lymphatic vessels, such as lymphatic endothelial hyaluronan receptor 1 and podoplanin, correlated with peritoneal thickness and UFF in human peritoneal membrane tissues [35,37,53]. These findings suggest that lymphangiogenesis was associated with peritoneal fibrosis in human PD patients. Both a chlorhexidine gluconate-induced model and methylglyoxal-induced peritoneal injury model indicate that lymph absorption was increased and inhibition of lymphangiogenesis resulted in an increase in drained PDF [35,37]. Inhibition of TGF-β suppressed VEGF-C and lymphangiogenesis in a chlorhexidine gluconate-induced model [53]. In cultured mesothelial cells and macrophages, TGF-β induced VEGF-C [53]. Such results suggest possible TGF-β–VEGF-C pathways for promoting lymphangiogenesis during peritoneal fibrosis.

### 3.4. Changes to Peritoneal Membrane Transport in PD

Four types of UFF can occur. Type 1 is the most common type, resulting from a rapid decline in osmotic conductance attributed to increases in peritoneal vascular surface area with angiogenesis. Type 2 involves dysfunction of aquaporin 1, a so-called ultra-small pore, in endothelial cells. Type 3 involves a hypo-permeable peritoneum. Adhesions arising under conditions of severe peritonitis are known to be a cause of this type of UFF. Type 4 involves increased lymph absorption as a cause of UFF [35,53].

The net UF volume is defined as transcapillary water transport minus lymphatic absorption [59] (Figure 2A). Free water transport with aquaporin 1 is driven by the crystalloid pressure gradient. In contrast, small pore fluid transport is driven by not only crystalloid, but also hydrostatic pressure, and is known to be affected by the degree of vasculopathy [59]. UFF is primarily due to declining transcapillary water transport and is associated with increased lymph absorption, as shown in animal models [35,40,46,53,60] (Figure 2B). Free water transport depends on the number of capillary vessels and probably on the volume of fibrosis. Incremental increases in the density of interstitial collagen explain the control of free water transport [26]. Reductions in both free water transport and small pore fluid transport seem to be observed in the majority of long-term PD patients. Improving transcapillary water transport and blocking lymphatic absorption are therefore potential targets to achieve a higher drainage volume in PD [35] (Figure 2C,D).

Recently, the International Society of Peritoneal Dialysis Special Series/Guideline classified the membrane dysfunction into three categories based on the underlying pathophysiology, allowing a better understanding of the pathogenesis [61]. These three types are as follows: (1) “High peritoneal solute transfer rate” due to membrane inflammation or neovascularization; (2) “Poor intrinsic ultrafiltration”, potentially due to genetic determinants including aquaporin 1; and (3) “Acquired intrinsic ultrafiltration insufficiency” due to structural changes in the interstitium during the development of peritoneal fibrosis, often associated with a fast peritoneal solute transfer rate. In this condition, an increase in the density of fibrosis increases the mechanical barrier, leading to increased resistance to water transfer [26]. Increased lymphatic absorption is not included in the peritoneal membrane dysfunction.

## 4. Roles of Cytokines and Growth Factors in Peritoneal Inflammation and Fibrosis in PD

Episodes of peritonitis and other stimuli including poor biocompatibility of dialysate cause peritoneal inflammation, which can induce fibrosis. Many cytokines induce fibrogenic growth factors, and crosstalk occurs between macrophages and fibroblasts.

### 4.1. Inflammation

Several inflammatory markers have been detected in dialysate from PD patients, such as interleukins (ILs), tumor necrosis factor (TNF)-α, and C-reactive protein, suggesting that inflammation is a trigger of peritoneal injury including fibrosis, angiogenesis, and lymphangiogenesis in PD. Multiple factors and pathways are involved in the development of peritoneal membrane inflammation in PD [62,63].

Plasma osmolality in pre-dialysis patients can reportedly rise as high as 323 mOsm/kg. Further, the peritoneal membrane is exposed to high glucose with high-osmolarity dialysate, and sodium is stored in the peritoneal membrane [48,64,65]. A local high-osmolarity environment activates the transcriptional factor nuclear factor of activated T cells 5, leading to increased levels of inflammatory chemokines and cytokines such as C-C motif chemokine ligand (CCL)-2 and IL-6 by mesothelial cells [65,66,67]. The PD catheter as well as bioincompatible dialysate may be involved in the induction of intraperitoneal inflammation and fibrosis [68] (Figure 1).

### 4.2. Macrophage Infiltration

The essential role of macrophage infiltration in the pathogenesis of peritoneal fibrosis has been demonstrated in several studies. Solute transport and UF function have been found to differ from the start of PD in a pathological study. Baseline D/P Cr was higher in patients with a higher density of CD68-positive macrophages in the peritoneum [33], indicating that inflammation is present in the uremic peritoneum before the initiation of dialysis treatment (Figure 1). This was then proved in uremic mouse models without direct peritoneal injury to the animals [64,69]. Increased peritoneal thickness and overexpression of TGF-β were also observed in this model, which may result in further peritoneal fibrosis and UFF.

Crosstalk occurs between macrophages and fibroblasts in a sodium hypochlorite-induced peritoneal injury mouse model. Resident macrophages with high expressions of CD11b and F4/80 disappeared and inflammatory macrophages were recruited from circulating monocytes. These inflammatory macrophages activated peritoneal fibroblasts via the chemokine CCL-17 [70]. Monocyte chemoattractant protein 1, the main chemokine regulating the recruit and infiltration of macrophages, was found to also be involved in the EMT process for mesothelial cells and directly increases ECM synthesis through its receptor CCR-2 [71].

Peritoneal fibrosis was suppressed in macrophage-depleted mice. M1 macrophage reperfusion induced ECM deposition in association with functional declines in UF, suggesting that M1 macrophages play an important role in the development of fibrosis and membrane dysfunction [72]. In recent studies, chronic exposure to dialysate has been shown to induce loss of homeostatic and anti-inflammatory potential characteristics in peritoneal resident macrophages, accompanied by enhanced inflammatory responses to external stimuli. Peritoneal resident M2 macrophages play a role in reducing the cytokine storm in acute infections [73]. Further, peritoneal M2 macrophage-derived extracellular vesicles could be used as multitarget therapeutics [62,74].

### 4.3. Inflammatory Cytokines

According to the GLOBAL Fluid Study, higher levels of inflammatory cytokines including IL-6 and TNF-α were found in the peritoneal dialysate of patients who subsequently developed encapsulating peritoneal sclerosis (EPS) [75]. IL-6, a well-known pro-inflammatory cytokine, plays a key role in mononuclear cell infiltration, leukocyte recruitment, and chemokine secretion. Two signaling pathways lead to IL-6: a classic pathway via the membrane-bound IL-6 receptor (IL-6R); and a trans-signaling pathway via soluble IL-6R and membrane-bound glycoprotein 130 in cells that do not express IL-6R [76]. IL-6 was found to directly activate transcription factor signal transducer and activator of transcription (STAT)3 via IL-6R and therefore activate the Janus kinase/signal transducer and activator of transcription (JAK/STAT) pathway. STAT3 was also found to be upregulated in long-term PD patients. EMT was observed to be promoted by IL-6 treatment via the activation of JAK2/STAT3 signaling in HPMCs [77]. In another in vitro study, treatment using IL-6 combined with soluble IL-6R stimulated VEGF-A expression in HPMCs, which was not observed with treatment using only either IL-6 or soluble IL-6R. This suggests that IL-6 trans-signaling plays the main role in this process. Transcriptional mechanisms involving STAT3 and SP4 were found downstream of this pathway. In a uremic pre-PD mouse model, blockade of IL-6 by the MR16-1 monoclonal antibody alleviated peritoneal angiogenesis and lymphangiogenesis, as well as cardiac fibrosis [65,78].

In addition to IL-6, in vitro experiments showed that high glucose levels induced the expression of HIF-1α via STAT3 signaling, along with higher expression of profibrotic markers such as collagen I, fibronectin, and α-smooth muscle actin in mesothelial cells [79].

Th17 lymphocytes are highly pro-inflammatory and are involved in various autoimmune diseases, while regulatory T lymphocytes are anti-inflammatory [80,81]. IL-6 and TGF-β are the main cytokines regulating the Th17/Treg balance. IL-6 promotes Th17 differentiation together with TGF-β, while Treg differentiation requires TGF-β and is inhibited by IL-6 [82]. Th17 is the main source of cytokine IL-17. Enhanced Th17-mediated inflammatory responses and/or IL-17 upregulated the expression of profibrotic factors and promoted the production of ECM proteins in animal models. Chronic exposure to PDF induced elevated levels of IL-17 in mice, suggesting a key role during peritoneal fibrosis in long-term PD. Recent studies have found that the leukocyte antigen CD69 also participates in the regulation of Th17/Treg balance [83]. CD69−/− transgenic mice showed increased numbers of Th17 cells, higher expression of IL-17, and enhanced peritoneal fibrosis compared to wild-type mice in a PDF-treated model. In addition, IL-17 blockade alleviated this process, suggesting that CD69 controls peritoneal fibrosis by regulating Th17 responses.

### 4.4. Inflammasomes

Inflammasomes are intracellular multiprotein complexes that control the release of the inflammatory cytokine IL-1β. The nucleotide-binding oligomerization domain-like receptor family pyrin domain-containing 3 inflammasome, which contains an apoptosis-associated speck-like protein containing a caspase recruitment domain (ASC), was found to be involved in inflammatory responses in sterile inflammatory diseases including cardiovascular diseases, type 2 diabetes, and metabolic syndrome [84]. Similarly, inflammasome activation in the peritoneal membrane during long-term exposure to PDF appears related to peritoneal deterioration. Deficiencies of nucleotide-binding oligomerization domain-like receptor family pyrin domain-containing 3, ASC, or IL-1β alleviated peritoneal inflammation and fibrosis in a methylglyoxal-induced peritoneal fibrosis murine model [85]. In addition, in another mouse model, ASC-positive and F4/80-positive cells that were found to be CD44-positive were shown to contribute to peritoneal fibrosis. CD44-positive macrophages in the circulation also count during this process [86].

### 4.5. Growth Factors for Development of Peritoneal Fibrosis (Table 1)

#### 4.5.1. TGF-β

TGF-β is a key mediator for PD-related peritoneal fibrosis. Exposure to high levels of glucose, a major osmotic agent in PD solution, upregulates TGF-β1 expression in HPMCs [87]. Elevated dialysate levels of TGF-β1 correlate with increased peritoneal transport rates in PD patients [88]. Overexpression of TGF-β1 by gene induction results in peritoneal thickening, collagen deposition, increased vascularization, and higher peritoneal transport rates in rats [89], suggesting that peritoneal fibrosis is linked to angiogenesis.

TGF-β1-blocking peptide suppresses mesothelial-to-mesenchymal transition (MMT) in cultured mesothelial cells treated with TGF-β1. This peptide also reduces peritoneal fibrosis and angiogenesis, improving impaired peritoneal function in a mouse model exposed to PDF [90]. Treatment with TGF-β receptor type 1 (TGF-βR-I) inhibitor suppresses morphological changes, cell migration, invasion, and EMT through the TGF-β/Suppressor of mothers against decapentaplegic (Smad) pathway in HPMCs exposed to TGF-β1. Administration of an orally available TGF-βR-I inhibitor attenuates peritoneal fibrosis in a murine model of chlorhexidine gluconate-induced peritoneal fibrosis [91]. TGF-β1 acts as an important inducer of angiogenesis and lymphangiogenesis via TGF-β–VEGF-A/C pathways, as described in Section 2.

**Table 1:** Factors involved in peritoneal fibrosis.

Target	Intervention	Model	Results	References
TGF-β	Gene induction	Rat	fibrosis↑, angiogenesis↑, peritoneal function↓	[89]
	Receptor inhibitor	Mouse, PD solution	fibrosis↓, angiogenesis↓, peritoneal function↑	[90]
	Receptor inhibitor	Rat, CG	fibrosis↓, VEGF-A/C↓, angiogenesis/lymphangiogenesis↓	[40,53]
CTGF	Monoclonal antibody	Mouse, CG	fibrosis↓, VEGF-A↓, angiogenesis↓	[92]
	Genetic deletion	Mouse, CG	fibrosis↓, inflammation↓, angiogenesis/lymphangiogenesis↓, peritoneal function↑	[34,93]
EGFR	Receptor inhibitor	Rat, CG	fibrosis↓, angiogenesis↓, TGF-β signaling↓	[94]
PDGF-B	Gene induction	Rat	VEGF-A↑, angiogenesis↑	[95]
Core fucosylation	shRNA	Rat, PD solution	fibrosis↓, TGF-β/PDGF/EGFR signaling↓	[96,97]
MMP9	Genetic deletion	Mouse, TGF-β1 gene transfer	VEGF-A↓, angiogenesis↓	[98]
MMP10	Genetic deletion	Mouse, CG	fibrosis↓, inflammation↓, VEGF-A↓, angiogenesis↓, peritoneal function↑	[99]
TG2	Genetic deletion	Mouse, CG	fibrosis↓, angiogenesis↓, EMT↓	[60]
Smad7	Gene induction	Rat, uremia, PD solution	fibrosis↓, peritoneal function↑, Smad2/3 activation↓	[100,101]
BMP7	Recombinant protein	Rat, uremia, CG	fibrosis↓, inflammation↓, angiogenesis↓, Smad3 pathway↓	[102]
HGF	Recombinant protein	Rat, PD solution	fibrosis↓, AGEs↓, TGF-β1↓, VEGF-A↓, angiogenesis↓	[103]

Abbreviations: TGF-β, transforming growth factor-β; PD, peritoneal dialysis; CG, chlorhexidine gluconate; VEGF, vascular endothelial growth factor; CTGF, connective tissue growth factor; EGFR, epidermal growth factor receptor; PDGF, platelet derived growth factor; shRNA, short hairpin RNA; MMP, matrix metalloproteinase; TG2, transglutaminase2; EMT, epithelial to mesenchymal transition; BMP, bone morphogenetic protein; HGF, hepatocyte growth factor; AGEs, advanced glycation endproducts. ↑: increase, ↓: decrease.

#### 4.5.2. CTGF

CTGF/CCN2 plays a major role in mediating ECM production during the progression of fibrosis, and its fibrogenic activities are mediated by other growth factors [104]. TGF-β derived from peritoneal fibroblasts and endothelial cells enhances CTGF expression in HPMCs [105]. CTGF expression is enhanced in peritoneal mesothelial cells and α-smooth muscle actin-positive fibroblasts in PD patients with UFF [106]. Intraperitoneal administration of an anti-CTGF monoclonal antibody reduces elevated CTGF levels, accumulation of myofibroblasts, number of CD31-positive vessels, and VEGF-A expression in a murine peritoneal fibrosis model exposed to chlorhexidine gluconate [92]. Inhibition of CTGF suppresses fibroblast proliferation, myofibroblast differentiation, MMT, and VEGF-A production in cultured fibroblasts and peritoneal mesothelial cells treated with TGF-β1 [92]. Similarly, genetic deletion of CTGF reduces peritoneal thickening, angiogenesis, and inflammation and normalizes increased peritoneal permeability in a chlorhexidine gluconate-treated mouse model [93]. CTGF is also involved in PD-related peritoneal lymphangiogenesis. Inhibition of CTGF partly reduces elevated VEGF-C expression in HPMCs treated with TGF-β1. Accordingly, CTGF inhibition suppresses peritoneal lymphangiogenesis in a chlorhexidine gluconate-treated mouse model [34].

#### 4.5.3. Other Fibrogenic Factors

Expression of epidermal growth factor receptor in peritoneal membrane is increased by chlorhexidine gluconate treatment in rats [94]. Intraperitoneal administration of gefitinib, an inhibitor of epidermal growth factor receptor, suppresses TGF-β1 expression, phosphorylation of Smad3, STAT3, and nuclear factor-κB (NF-κB), which leads to reduction in peritoneal fibrosis and a number of CD31-positive blood vessels in the model [94]. Treatment with gefitinib also inhibits phosphorylation of Smad3 and EMT in HPMCs exposed to TGF-β1 [94].

Peritoneal overexpression of platelet-derived growth factor B by adenovirus gene induction increases VEGF-A expression and promotes angiogenesis, but it does not upregulate TGF-β/Smad signaling or peritoneal fibrosis [95]. The angiogenic effects of platelet-derived growth factor B are independent of TGF-β signaling in mice [107].

Core fucosylation of N-glycans on TGF-β receptors is required for their receptor function [108]. Expression of core fucosylation is increased in a rat model of peritoneal fibrosis exposed to high-glucose dialysate. Inhibition of core fucosylation by α-(1,6)-fucosyltransferase (Fut8) short hairpin RNA inactivates TGF-β1 and platelet-derived growth factor signaling and attenuates peritoneal fibrosis in the model [96]. Inhibition of core fucosylation also suppresses the phosphorylation of STAT3 and NF-κB that results from the epidermal growth factor receptor signaling pathway in the rat model [97].

Matrix metalloproteinases (MMPs) are a family of proteinases involved in remodeling the ECM and cleaving cell surface proteins [109]. MMP2 expression in PD effluent correlates strongly with peritoneal solute transport rate and is expected to provide a useful biomarker of peritoneal injury [110]. Expression of MMP9 in HPMCs also correlates with higher peritoneal solute transport rates [98]. Overexpression of MMP9 in mouse peritoneum induces peritoneal thickening and angiogenesis through MMT [98]. Genetic deletion of MMP9 reduces VEGF-A expression and suppresses peritoneal angiogenesis in mice after gene transfer of TGF-β1. This effect is possibly due to reduced E-cadherin cleavage and suppression of β-catenin signaling [98]. MMP10 is increased in peritoneal macrophages and mesothelial cells in fibrotic peritoneum obtained from patients and model mice. Genetic deletion of MMP10 reduces peritoneal fibrosis, inflammation, VEGF-A expression, number of CD31-positive vessels, and elevated peritoneal solute transport rates in a mouse model exposed to chlorhexidine gluconate. The anti-inflammatory effects possibly result from inactivation of the NF-κB pathway [99].

Transglutaminase (TG)2 is a key enzyme in the pathogenesis of fibrosis through TGF-β signaling and matrix cross-linking mechanisms [111]. Genetic deletion of transglutaminase 2 decreases TGF-β expression and suppresses peritoneal fibrosis and angiogenesis in a chlorhexidine gluconate-induced murine model of peritoneal fibrosis associated with attenuated EMT [60].

### 4.6. Anti-Fibrogenic Factors

TGF-β is known to transduce signals via Smad- and non-Smad-dependent pathways. TGF-β receptor-induced phosphorylation of Smad2 and Smad3 is inhibited by Smad7 [112]. Increased Smad2/3 activation and decreased Smad7 expression is found in a peritoneal fibrosis model exposed to PD solution containing high concentrations of glucose in uremic rats [100,101]. Overexpression of Smad7 by gene transfer inhibits Smad2/3 activation, reduces peritoneal fibrosis, and improves impaired peritoneal function in the model [100,101]. The non-Smad signaling pathway reportedly showed activation of protein kinase C, c-Jun N-terminal kinase, extracellular signal-regulated kinase, and phosphatidylinositol-3-kinase activating the serine-/threonine-specific protein kinase. The Smad/non-Smad pathway is described in detail elsewhere [113].

Bone morphogenetic protein (BMP)7 counteracts TGF-β-mediated activity, thus suppressing fibrosis progression [114]. Exposure to high levels of glucose reduces bone morphogenetic protein 7 expression in HPMCs [115]. Increased expression of bone morphogenetic protein 7 by supplementation with recombinant protein or gene transfer suppresses EMT in HPMCs exposed to high concentrations of glucose [115]. Intraperitoneal administration of bone morphogenetic protein 7 inhibits peritoneal Smad3 expression and its phosphorylation, peritoneal fibrosis, inflammation, and angiogenesis in a chlorhexidine gluconate-treated model in uremic rats [102].

Hepatocyte growth factor is a key anti-fibrogenic factor for preventing organ fibrosis. Treatment with hepatocyte growth factor prevents EMT in HPMCs exposed to high levels of glucose [115]. Treatment with hepatocyte growth factor prevents peritoneal thickening and ameliorates accumulation of AGEs, expression of TGF-β1, type 1 collagen, VEGF-A, and the number of blood vessels in a rat model exposed to PD solution [103]. Similar anti-fibrogenic effects of hepatocyte growth factor were also found in mouse models of peritoneal fibrosis induced by chlorhexidine gluconate [116,117] and methylglyoxal [118], which is a toxic GDP.

## 5. Roles of Complement Activation in Peritoneal Inflammation and Fibrosis in PD

### 5.1. Complement System in the Process of Fibrosis and the Fibrinolytic System

Little direct evidence has been accumulated linking fibrosis and the complement system. However, the complement system is at least indirectly related to fibrinogenesis and the fibrinolytic system. Basically, under controlled activation of the complement system, activation products of the complement system work to clear immune complexes and broken, disused fragments, to protect the host from attacks by invasive microorganisms and to eliminate malignant cells as a first line of defense [119,120]. Activation of the complement system is tightly controlled with numerous complement regulatory proteins in both fluid and solid phases (Table 2) [119]. However, uncontrolled activation of the complement system can occur with injury to tissues involved in the process of enhancing the complement activation system and/or impairment of membrane complement regulators [119,120]. Fibrosis also develops to repair those injured tissues. This is one way to explain the relationship between fibrosis and the complement system and may be the main story. During activation of the complement system, production of large amounts of C3a, C4a, and C5a and formation of C5b-9 induces inflammation and the development of tissue injuries under conditions of excessive complement activation. As a result, tissue fibrosis develops in various tissues such as the lungs, liver, and heart [121,122,123,124]. To prevent retinal fibrosis, VEGF-associated progression of atherosclerosis might be regulated by complement regulators in retinal micro-vasculopathy [125]. Concerning the lectin pathway, Liu et al. reported that extracellular vesicles with mannose-associated serine protease 1 from hepatocytes induce liver fibrosis through p38 mitogen-activated protein kinase (MAPK)/Atf2 signaling [126].

In contrast, the possibility of recovery from fibrotic processes has also been reported. For example, C5 might work to decrease the accumulation of CD45+ cells and suppress MMP9 production according to a study using C5-deficient mice [127]. Both C3a and C5a, as anaphylatoxins produced through an alternative pathway (AP) and/or the formation of complement, might work to restore fibrosis by proceeding through changes in polarization from M2 macrophages to M1 macrophages [128,129].

The fibrinolytic system is associated with fibrinogenesis and also the coagulation system. The complement system also cross-reacts with the coagulation system. For example, C1 inhibitor, a fluid-phase complement regulator, was also discovered to play roles in the coagulation system as a C1 inhibitor. The complement system might be related to fibrinogenesis through the fibrinolytic system. From the perspective of the complement system, concerning the fibrinolytic system and fibrosis, relationships among thrombin, thrombomodulin, and thrombin-activatable fibrinolysis inhibitor were shown in the late 1990s [130]. Thrombin-activatable fibrinolysis inhibitor is also known as “carboxypeptidase R” in the complement system (Table 2) and has been shown to strongly suppress effects against fibrinolysis in thrombin-activatable fibrinolysis inhibitor-deficient mice [131].

**Table 2:** Complement regulatory proteins in fluid and solid phases in humans.

Molecule	Molecular Weight	Regulatory Point	Distribution in Peritoneum	References
Fluid phase				
C1 inhibitor (C1-INH, C1 inactivator)	~80 kDa,	CP *	N/A *****	N/A
	~100 kDa			
factor I (CFI)	α chain 50 kDa,	C3 convertase	N/A	N/A
	β chain 38 kDa			
C4-binding protein (C4BP)	~ 500 kDa	CP	N/A	N/A
factor H (CFH)	~155 kDa	AP **, C3 convertase	N/A	N/A
vitronectin (S-protein, epibolin, plasminogen activator inhibitor (PAI)-1-binding protein)	~75 kDa	TP ***	N/A	N/A
clusterin	80 kDa	TP	N/A	N/A
carboxypeptidase N	50 kDa, 85 kDa	anaphylatoxin	N/A	N/A
carboxypeptidase R **** (carboxypeptidase B)	60 kDa	anaphylatoxin	N/A	N/A
Solid phase				
membrane cofactor protein (MCP, CD46)	~65 kDa, ~55 kDa	C3 convertase	Mesothelial cells	[132,133]
			Endothelial cells	
decay accelerating factor (DAF, CD55)	~70 kDa	C3 convertase	Mesothelial cells	[132,133]
			Endothelial cells	
complement receptor 1 (CD35)	160-over 250 kDa	C3 convertase	N/A	N/A
CD59	~20 kDa	TP	Mesothelial cells	[132,133]
			Endothelial cells	

*, the classical pathway in the complement system; **, alternative pathway; ***, the terminal pathway in the complement system; ****, thrombin-activatable fibrinolysis inhibitor (TAFI), *****, not available.

Conversely, some complement regulatory proteins could play roles in fibrinogenic and/or fibrinolytic processes. Vitronectin works as a fluid-phase membrane regulatory protein and shows brief activity to prevent the formation of the membrane attack complex (C5b-9) in the complement system, but the binding of vitronectin to collagen and plasminogen activator inhibitor 1 might be associated with the prevention of fibrinolytic activity [134]. Induction of alveolar decay acceleration factor (CD55), a membrane complement regulatory protein (CReg), may also suppress the progression of fibrosis in the lungs [135]. In contrast, VEGF might control expression of CRegs because VEGF is known to induce retinal expression of complement factor H, another complement regulator, through VEGFR2/PKC-α/CREB signaling [125].

### 5.2. Peritoneal Fibrosis Associated with Complement Activation

An early report associated with the complement system in PD was published in the early 1980s. In 1981, Blumenkrantz et al. reported protein leakage of C3 and C4 as complement components into the peritoneal cavity [136]. Activation of the complement system might occur continuously in the peritoneal cavity of PD patients, as a 1993 study measured complement activation products in PD patients [137]. In the peritoneum as in other tissues, complement components are mainly delivered from the systemic circulation.

Although most complement components are produced in the liver, local production of complement components is seen in the peritoneum. In fact, local production of complement components such as C3 and C4 by mesothelial cells was also reported in 1991 [138]. Regulation of the complement system is important in the host because of the effects of eliminating foreign bodies and host tissues themselves, in so-called “double-edged roles” [120]. As we reported, expression of CRegs was observed in the peritoneum and peritoneal injuries developed autologously with neutralization of Creg function in the peritoneum [139]. We showed that peritoneal expression of CD55 correlated inversely with soluble C5b-9 as a terminal pathway product using HPMCs from PD effluent. Some unknown factors likely affected the expression of CRegs after reaching a threshold degree of complement activation [132]. In peritoneal tissues obtained from PD patients, Kitterer et al. showed similar data for CD55 expression [133]. Because modification of CD59 as a Creg was also shown in an in vitro study, suggesting enhancement of atherosclerosis [140], damage to the CD59 protein caused by long-term glucose exposure might affect PD patients. Those data suggest that expression and functional activation of CRegs might sometimes occur in the peritoneum and that Creg instability might promote a loss of balance between complement activation and CRegs.

As we previously reported [141,142,143], excessive activation of the complement system can lead to the development of peritoneal injuries accompanied by peritoneal fibrosis. As peritonitis caused by fungus is well known to cause severe intestinal adhesion, peritoneal fibrosis, and also EPS, we induced severe peritonitis by intraperitoneal administration of fungus-derived zymosan in rats with pre-scraping against the parietal peritoneum [141,142]. In this unique animal model, severe peritoneal thickness and fibrinogen deposition were observed in the peritoneum. In the peritoneum of PD patients with peritonitis caused by fungal infection, we observed similar pathological changes accompanied by decreased peritoneal expression of CRegs and massive deposition of complement activation products in some cases [144]. In particular, excessive complement activation could be induced with the impairment of complement regulators such as peritoneal injuries due to peritonitis and/or exposure to PDF [139,145]. In experimental animal models of peritonitis associated with fungal infection, the alternative pathway might be overactivated and lead to the development of peritoneal injuries accompanied by progression of peritoneal fibrosis [141,142]. With these results, we observed fibrogenesis accompanied by peritoneal inflammation.

Under the circumstance of PD therapy, peritoneal mesothelial cells are frequently exposed to low pH in acid-based conventional PDF and high-glucose PDF. In the 1990s, high-glucose PDF was shown to induce C3 production by mesothelial cells in a main complement component of the alternative pathway [146]. We showed increases in complement activation products such as sC5b-9 in PDF, which might induce D/P Cr increases [132]. Such changes might be related to peritoneal fibrosis through complement activation.

Bartosova et al. showed that C1q, C3d, terminal complement complex, and phosphorylated SMAD2/3, a downstream effector of TGF-β in arterioles of the omentum and parietal peritoneum, as well as deposition of C1q and C5b-9, correlated with the level of exposure to dialytic glucose, the abundance of phosphorylated SMAD2/3, and degree of vasculopathy. They concluded that PDF activates arteriolar complement and TGF-β signaling, which correlate quantitatively with the severity of arteriolar vasculopathy, including fibrotic changes [147].

Until now, direct relationships between the complement system and fibrinogenesis have remained unclear. However, since the complement system is always ready to be activated in the alternative pathway through “tick-over” and has an “amplification loop” for activation, complement activation may directly or indirectly lead to the development of fibrosis in the peritoneum.

Recently, C1 inhibitors to neutralize C1 and coagulation systems, eculizumab, ravulizumab, and crovalimab as anti-C5 antibodies to neutralize terminal pathways, and anti-C1s antibody to prevent activation of the classical pathways have been on the clinical stage [148]. Numerous other anti-complement agents are being developed from pre-clinically to clinical phase 3 trials [148,149]. In a series of our past reports, the efficacies of C1-inhibitor, C5a antagonist peptide, and anti-C5b-7 neutralizing antibody were shown to prevent the development of peritoneal fibrosis associated with peritonitis in addition to first-generation anti-complement agents such soluble complement receptor 1 and a C5a receptor antagonist [139,141,142,143,150]. We expect that anti-complement therapy may become a prime choice among therapeutic strategies to prevent or improve peritoneal injuries in the future.

## 6. Peritoneal Pathological Findings in EPS and Effects of PD Solutions on Pathologies of the Peritoneal Membrane

This section covers characteristic features of EPS and differences in peritoneal injuries induced by conventional PDF and low-GDP pH-neutral solutions, and possible predictors of EPS.

### 6.1. Peritoneal Pathological Changes of EPS (Table 3A)

EPS is a rare but significant complication of PD therapy. Pathologically, EPS is characterized by refractory and recurrent bowel obstruction due to fibrous capsules [151,152].

Known risk factors for EPS include long-term dialysis [151,153,154], peritonitis [155,156], and renal transplantation [155,157,158]. The characteristics of peritoneal tissue in patients with EPS have been studied in detail. Peritoneal fibrosis in PD can be pathologically classified into two categories: simple peritoneal sclerosis; and EPS. Simple peritoneal sclerosis carries a degree of risk but does not necessarily lead to EPS, and a second trigger in the presence of peritoneal deterioration called simple peritoneal sclerosis may be needed to lead to EPS [159].

**Table 3:** Pathological findings in the peritoneal membrane. (**A**) Pathological findings in the peritoneal membrane with and without EPS. (**B**) Differences in pathology of the peritoneal membrane treated with pH-neutral PD solution and conventional PDF.

**(** **A)**						
	**Number of Patients**	**PD Duration**	**Peritoneal Thickness**	**Vasculopathy**	**Vessel Density**	**Year**
Garosi et al. [160]	Non-EPS (N = 180) EPS (N = 39)	on PD for 6 months to 12 years	75 (10–70) μm 750 (250–4000) μm (*p* < 0.01)	Prevalence of vasculopathy 11% 100% (*p* < 0.01)		2005
Alscher et al. [161]	Non-EP: (N = 26) EPS (N = 9)	19 ± 28 months 93 ± 27 months	Peritoneal thickness was higher in EPS group. (*p* < 0.001)	No significant difference.	No significant difference.	2007
Sherif et al. [162]	Non-EPS (N = 13) EPS (N = 12)	106.3 ± 20.9 months 116.3 ± 38.2 months	527.2 ± 457.4 μm 552.3 ± 331.9 μm (*p* = 0.5)	No significant differences.	36.6 ± 27.1 /mm^2^ 37.2 ± 21.9 /mm^2^ (*p* = 0.6)	2008
Braun et al. [163]	Non-EPS (N = 27) EPS (N = 31)	37.6 ± 38.0 months 77.5 ± 41.2 months	602.9 μm 1132.5 μm (*p* = 0.0031)	Prevalence of vasculopathy 26% 35% (*p* = 0.40)	41% 52% (*p* = 0.61)	2012
Morelle et al. [26]	Non-EPS (N = 28) EPS (N = 7)	57.8 ± 7.6 months 55.9 ± 4.2 months	Submethotelial thickness was higher in EPS group (*p* < 0.01).	Vasculopathy grade was higher in EPS group (*p* < 0.05).	Higher in EPS group (*p* < 0.01).	2015
Tawada et al. [164]	EPS-conventional solution (N = 28) EPS-pH neutral solution (N = 7)	132.9 (105.4–157.2) months 63.1 (29.8–83.1) months	528.4 (359.3–798.4) μm 348.1 (314.6–812.0) μm (*p* = 0.4).	L/V ratio 0.00 (0.00–0.51) 0.67 (0.64–0.78) (<0.001).	12.5 (5.7–23.7)/mm 29.0 (12.3–56.0)/mm (*p* < 0.05).	2021
**(B)**						
	**Number of Patients**	**PD Duration**	**Peritoneal Thickness**	**Vasculopathy**	**Vessel Density**	**Year**
Kawanishi et al. [165]	Conventional solution (N = 12) pH-neutral solution (N = 12)	57.0 ± 5.97 months 51.9 ± 5.9 months	482.5 ± 24.3 μm 281.4 ± 34.4 μm (*p* < 0.05)	L/V 0.50 ± 0.03 0.86 ± 0.03 (*p* < 0.05)	Vessel density 30.6 ± 3.5 /mm^2^ 90.4 ± 3.3 /mm^2^ (*p* < 0.05)	2013
Hamada et al. [166]	Conventional solution (N = 80) pH-neutral solution (N = 61)	62.5 ± 43.3 months 33.6 ± 23.1 months	No significant difference for at least 60 months.	Patency in neutral group was significantly higher compared to that in acidic group.		2015
del Peso et al. [167]	Conventional solution (N = 23) pH-neutral solution (N = 23)	24.2 ± 18 months 22.7 ± 16 months	Prevalence of fibrosis 69.6% 47.8% (*p* = 0.13)	Prevalence of vasculopathy 30.4% 4.3% (*p* = 0.02)		2016
Tawada et al. [45]	Conventional solution (N = 54) pH-neutral solution (N = 73)	102 (75.0–132.0) months 44 (19.0–72.0) months	375.0 (274.06–602.00) μm 244.0 (154.68–390.25) μm (*p* < 0.001)	L/V 0.50 ± 0.17 0.76 ± 0.06 (*p* < 0.001)	CD31-vessels (number/mm) 14.0 (10.28–17.07) 12.4 (7.68–17.18) (*p* = 0.354)	2019
Sugiyama et al. [168]	Conventional solution (N = 11) pH-neutral solution (N = 11)	111.9 ± 22.1 month 98.6 ± 18.2 months	395.3 (351.1–516.4) μm 281.4 (150.1–419.3) μm (not significant)	L/V 0.43 ± 0.18 0.73 ± 0.08 (*p* < 0.05)	Vessels (number/mm) 16.3 ± 6.1 11.5 ± 3.0 (*p* < 0.05)	2022

Garosi et al. described the findings of peritoneal pathology in EPS [169]. They described peritoneal thickening, peritoneal ossification, vascular alterations, and macrophagic giant cells in fibrin-rich exudate. In addition, severe vasculopathy and occlusion of vessels are hallmarks of EPS [160]. One report found that patients with EPS displayed significantly greater peritoneal thickening and vasculopathy at the highest level, but no significant difference in vascular damage compared to PD patients without EPS [161]. Honda et al. compared 12 patients with EPS and found that the most prominent histological features of EPS were fibrin deposition and fibroblast swelling [170]. Similarly, Sherif et al. found significant differences in fibrin deposition and degenerated thickness of the submesothelial layer. No significant differences were found in compact zone thickness, vessel density, or vasculopathy [162]. In an analysis of seven patients with EPS, decreased osmotic conductance was a predictor of EPS, and was primarily attributed to alterations to the collagen matrix in the peritoneal membrane [26]. In this report, the vasculopathy grade was higher in the EPS group. Unfortunately, those analyses were conducted in a small cohort.

Braun et al. analyzed the pathology of 31 patients with EPS. They found that podoplanin expression, fibroblast-like cells, mesothelial denudation, calcification, acellular areas, and fibrin deposits were characteristic of EPS [163]. The same group noted that areas diffusely positive for podoplanin are found in many cases of EPS and may be related to EPS risk factors [171]. They compared characteristic patterns of podoplanin staining by classifying EPS into four categories [172]. Our group examined the new membrane capsule of EPS. Although new membrane can be seen even in non-EPS cases, staining for fibrin and podoplanin can distinguish the stage of EPS, such as early exudative changes with fibrin deposition, proliferative and fibrosing changes with podoplanin-positive fibrosis, fibrosing changes with fibrin deposition, podoplanin-positive fibroblast-like cells, and adhesive and fibrous scar formation [173]. Such staging may be clinically related to the pre-EPS period, inflammatory period, encapsulating period, or period of suppression of inflammation with scarring [174].

We recently examined peritoneal EPS tissues obtained at enterolysis surgery from 174 patients, with 165 receiving conventional PDF and nine receiving pH-neutral solution. Patients using the conventional PDF exhibited more vascular damage with a lower L/V ratio. EPS recurrence occurred in cases with more severe vascular damage [164]. In addition, we examined which pathological findings could be used as predictors of EPS using peritoneal membrane pathology determined at cessation of PD and removal of the catheter. Vascular injury with severe vasculopathy and fibrin deposition could offer pathologic predictors of EPS (13). These findings seem to be driven by endothelial injury resulting from the bioincompatibility of conventional PDF. Such findings are consistent with the leak-to-response hypothesis proposed by Nakayama, in which EPS is caused by severe vascular damage in long-term PD patients, leading to fibrin leaching that covers the entire peritoneum [175]. In EPS cases treated with pH-neutral solution, peritonitis represents an important cause of EPS via inflammation-induced adhesion processes [164].

### 6.2. Effects of PD Solution in the Peritoneal Membrane (Table 3B)

In the 2000s, a low-GDP, pH-neutral PD solution was developed as a more biocompatible option. With this development, differences in peritoneal tissue injury between low-GDP, pH-neutral solution, and conventional PDF were reported. A study of 11 PD patients on pH-neutral solutions showed no correlation between peritoneal thickening, vascular damage, and PD duration, with minimal findings for each [176].

Various studies have compared peritoneal tissue changes between conventional and pH-neutral PDFs. In 2013, comparisons were made for 12 patients with a PD history of approximately 55 months using a pH-neutral solution with low GDPs or a conventional PDF containing high GDPs. The group using pH-neutral solution showed improvements in peritoneal thickening, vascular damage, and deposition of AGEs. The number of blood vessels was increased with neutral solution [165]. Similarly, Hamada et al. studied 61 patients using pH-neutral PD solutions [166], and del Peso et al. compared 23 pH-neutral biocompatible PD solutions with 23 conventional PD solutions. No differences were seen in peritoneal thickening, but vascular injury was mild in patients using pH-neutral solution [167].

We analyzed 54 patients with conventional PDF and 73 patients with pH-neutral PDF and found that peritoneal thickening and vascular damage were improved and AGE deposition was reduced in the pH-neutral solution group. In addition, the L/V ratio decreased significantly over time in the conventional PDF group, whereas no such change was observed in the pH-neutral solution group. D/P Cr and L/V ratios correlated negatively with the duration of PD treatment in the conventional PDF group, but not in the pH-neutral solution group [45]. We reported that heparan sulfate and *Ulex europaeus* agglutinin lectin-1-binding sugar chains were more reduced in the conventional PDF group compared to the pH-neutral solution group [168], suggesting that components of the endothelial glycocalyx are involved in peritoneal transport in PD, and are better preserved with the use of pH-neutral PDF.

Given such findings, pH-neutral dialysate has been shown to reduce peritoneal damage compared to conventional PDF, preventing peritoneal deterioration as the first factor in the two-hit theory of EPS [159]. In fact, our study of EPS revealed that the majority of triggers for EPS with acid dialysate were long-term dialysis, whereas most triggers with pH-neutral solution dialysate were peritonitis [164]. Further, Kawanishi et al. [154] reported that the incidence of EPS in Japan was 2.5% using conventional PDF, dropping to 1.0% after the introduction of pH-neutral solution [177]. The use of neutral dialysate is expected to allow safer long-term PD.

In contrast, benefits in terms of preservation of peritoneal integrity (including reductions in inflammation, EMT, and fibroblast activation) are much less apparent in children [178]. In a large pediatric study, the peritoneal membranes of 82 patients with PD were examined after the use of pH-neutral, low-GDP dialysate. The number of vessels correlated with the peritoneal small-solute transport rate. In children on PD, pH-neutral solution containing low levels of GDPs induces early peritoneal inflammation, angiogenesis, fibroblast activation, and EMT, differing markedly from the situation in adults [178].

Studies of peritoneal tissues are expected to elucidate the biological response mechanisms of PD and thereby facilitate the development of PD solutions offering better biocompatibility. Response to pH-neutral solution seems to differ between adults and children. Further study is clearly needed to clarify these differences and the underlying causes.

## 7. Prevention and Summary

Prevention of peritonitis is the most important step toward preserving peritoneal membrane function. Glucose-sparing strategies and dialysate with better biocompatibility would be useful for reducing damage to the peritoneal membrane. Low-GDP, pH-neutral solutions appear preferable, at least for adults [179,180]. Avoiding a high-salt diet is also important. Please refer to the related articles for other factors including regulation of epigenetic genes, microRNA, glycolytic and pyruvate metabolism, and pseudohypoxia [44,181,182,183].

Recently, several drugs including tamoxifen, pirfenidone, nintedanib, TGF-β inhibitors, CTGF inhibitors, AGE inhibitors, bioactive agents from *Astragalus membranaceus*, baicalein, silymarin, and chemokine (C-C motif) ligand 8 inhibitor have been reported to reduce peritoneal fibrosis in animal models [81,93,184,185,186,187,188,189,190]. Other therapeutic trials targeting mediators of peritoneal membrane changes have also been attempted (Figure 1). At least some of these drugs will hopefully become available for clinical use.

A more complete understanding of the pathophysiology underlying peritoneal fibrosis and peritoneal membrane dysfunction may lead to better preservation of peritoneal membrane function and fluid volume control. In addition, new PD dialysates and pharmacological agents could be developed based on these findings. Continued studies will hopefully lead to improvements in technical survival, survival, and quality of life for PD patients.

## Abbreviations

AGEs	advanced glycation end products
AQP1	aquaporin-1
CCL2	C-C motif chemokine ligand 2
CTGF/CCN2	connective tissue growth factor/cellular communication network factor 2
D/P Cr	dialysate-to-plasma ratio of creatinine
ECM	extracellular matrix
EMT	epithelial–mesenchymal transition
EPS	encapsulating peritoneal sclerosis
GDPs	glucose degradation products
HPMCs	human peritoneal mesothelial cells
IL	interleukin
L/V ratio	ratio of luminal diameter (L) to vessel diameter (V)
MAC	membrane attack complex
MMPs	matrix metalloproteinases
PCV	post-capillary venule
PD	peritoneal dialysis
PDF	peritoneal dialysis fluid
pH-neutral solution	low-GDP pH-neutral solution
Smad	Suppressor of mothers against decapentaplegic
STAT3	signal transducers and activators of transcription 3
TGF-β	transforming growth factor-β
TNF-α	tumor necrosis factor α
UFF	ultrafiltration failure
VEGF	vascular endothelial growth factor
